# A Systematic Literature Review on the Composition, Health Impacts, and Regulatory Dynamics of Vaping

**DOI:** 10.7759/cureus.66068

**Published:** 2024-08-03

**Authors:** Annayat Ghuman, Priyanka Choudhary, Jyoti Kasana, Sumana Kumar, Hemant Sawhney, Ramdas Bhat, Ritik Kashwani

**Affiliations:** 1 Department of Oral and Maxillofacial Surgery, Baba Farid University of Health Sciences, Faridkot, IND; 2 Department of Oral Pathology and Microbiology, Poornima Sethi Multi Speciality Hospital, New Delhi, IND; 3 Department of Periodontology, North Delhi Municipal Corporation Medical College, Hindu Rao Hospital, New Delhi, IND; 4 Department of Microbiology, School of Life Sciences, JSS Academy of Higher Education and Research, Mysuru, IND; 5 Department of Oral Medicine and Radiology, School of Dental Sciences, Greater Noida, IND; 6 Department of Pharmacology, Rajiv Gandhi University of Health Sciences, Mangalore, IND

**Keywords:** vegetable glycerin, vaping, propylene glycol, flavoring agents, electronic cigarettes

## Abstract

This comprehensive review deals with the multifaceted aspects of electronic cigarettes (e-cigarettes), examining their composition, health implications, regulatory challenges, and market dynamics. E-cigarettes, also known as vaping devices, function by warming a solution of liquid containing flavors, nicotine, and various other compounds to produce an aerosol for users to inhale. This review underscores the evolution and widespread adoption of e-cigarettes since their introduction in 2003, highlighting their appeal as alternatives to traditional tobacco smoking. The essential parts of e-cigarettes are the battery, heating element, e-liquid (or e-juice), and mouthpiece. Propylene glycol and vegetable glycerin are common ingredients in e-liquids, along with nicotine and other flavors. Concerns over the health impacts of e-cigarettes have grown, particularly in light of incidents like the e-cigarette or vaping-associated lung injury outbreak in 2019 linked to vaping-associated lung injuries. Evidence suggests that while e-cigarettes may pose fewer risks than conventional cigarettes, they are not without health consequences, including potential respiratory and cardiovascular effects. Regulatory efforts worldwide have struggled to keep pace with the rapid evolution of e-cigarettes, exacerbated by their diverse flavors and marketing strategies that appeal to youth. The review discusses global regulatory responses, including bans and restrictions, to curb youth uptake and address public health concerns. Furthermore, the rise of a black market for e-cigarettes poses additional challenges to effective regulation and tobacco control efforts. In conclusion, while e-cigarettes offer potential harm reduction benefits for adult smokers seeking alternatives to traditional tobacco products, their widespread availability and evolving landscape necessitate vigilant regulatory oversight to protect public health, especially among youth. Future research should continue to explore the long-term health impacts and efficacy of e-cigarettes as smoking elimination aids, informing evidence-based policies and interventions.

## Introduction and background

Electronic cigarettes (e-cigarettes) are battery-operated gadgets that generate an inhaled aerosol by warming liquid comprising flavors, nicotine, and various chemicals of other kinds [1]. The word vaping refers to the idea that expelled smoke is water vapor. It is made up of very small chemical particles. The vaping device comprises a mouthpiece, a power source, an e-liquid cartridge, and a heating component. When the gadget is being used, the battery warms the heating component, which transforms the e-liquid contents into an aerosol that is taken in and breathed out. Vaping products comprise e-cigarettes, vape pens, and personal vaporizers (or at times known as "multiple organ dysfunction syndrome"). The e-liquid in vaporizer equipment typically consists of a propylene glycol (PG) or vegetable glycerin (VG)-based liquid containing nicotine, flavorings, and other chemicals and metals, but it does not contain tobacco [2].

In the latter months of 2019, the Centers for Disease Control and Prevention (CDC) began investigating a significant spike in admissions to hospitals due to vaping devices. Patients reported a wide range of symptoms related to breathing, including shortness of breath, coughing, and chest tightness. They all had one thing in common: they had been using vaping devices during the previous three months. The ailment was characterized as "e-cigarette, or vaping, product use-associated lung injury" or e-cigarette or vaping-associated lung injury (EVALI). By February 2020, the CDC had documented more than 2,800 hospitalizations and 68 deaths from EVALI [3]. EVALI stands for e-cigarette or vaping-related lung damage. It was initially referred to as vaping-associated pulmonary disease. The new categorization comes in response to an increased number of serious lung illness cases connected to the use of e-cigarettes and vaping devices, with the first case recorded in 2019. We are constantly learning about this condition; therefore, the nomenclature may continue to evolve [4].

The e-cigarette was first produced in China in 2003 and has since become widely accessible. It is made up of several components, including a battery, a microchip, a red light-emitting diode that mimics the burning tip of a cigarette, a cartridge that holds the liquid (PG in water), and a vaporization chamber that creates an aerosol that mimics cigarette smoke [5]. The assumption among consumers that utilizing electronic nicotine delivery systems (ENDS), often known as e-cigarettes or "vaping," is less dangerous to human health than smoking traditional tobacco cigarettes has contributed to the sharp rise in the use of e-cigarettes. ENDS are becoming more and more popular since they are frequently utilized as a smoking cessation aid. The potential harmful consequences of ENDS and e-liquids, often made of PG, VG, nicotine, and flavoring compounds, are not fully understood due to the novelty of ENDS and their variety. On the other hand, using ENDS is linked to detrimental health outcomes like organ damage, oxidative stress, and inflammation [6].

More than 4,000 compounds with various immunomodulatory and other effects on the lungs are present in traditional cigarette smoke. E-cigarettes are touted as being less dangerous compared to their effects. However, according to the limited number of published in vitro or in vivo investigations, e-cigarette vapor appears to be harmful to both experimental animals and cultured cells [7].

This study aimed to investigate if e-cigarette aerosols containing both PG and VG in equal ratios affect the mucociliary system and inflammation in the airway epithelium, which is relevant to e-cigarette usage in real life. Researchers discovered that in vitro primary human bronchial epithelial cells are affected by PG/VG aerosols in terms of cystic fibrosis transmembrane conductance regulator and big potassium channel function. PG/VG aerosols may have caused goblet cell hyperplasia by further decreasing ciliary beating and upregulating MUC5AC expression. In sheep airways in vivo, PG/VG e-cigarette aerosols also raised matrix metalloproteinase-9 activity and mucus concentration. Taken together, these findings prove the detrimental impacts of PG and VG on the respiratory system [8].

## Review

Methodology

Search Strategy

A complete literature search was carried out utilizing the electronic databases PubMed, Google Scholar, and Web of Science. The search technique includes keywords and Medical Subject Headings phrases relating to the review issue, such as "vape," "EVALI," "treatment," "diagnosis," and "outcomes." Boolean operators (AND, OR) were employed to combine search phrases and narrow the results.

Inclusion Criteria

The review criteria included peer-reviewed papers published in English, ensuring the reliability and accessibility of the information. The studies were conducted in humans, providing directly applicable insights. Only articles published within the last 10 years were considered, ensuring the relevance and timeliness of the data. Additionally, the studies needed to focus specifically on the specified review topic to maintain a clear and targeted scope.

Exclusion Criteria

The review excluded non-peer-reviewed publications, such as editorials, letters, and case reports, to ensure the credibility and rigor of the included studies. Additionally, studies that were not directly related to the review topic were omitted to maintain a focused analysis. Publications in languages other than English were also excluded to ensure consistency and accessibility.

Data Extraction

Data from relevant studies were retrieved using a standardized data extraction template, ensuring consistency and thoroughness. For each study, the following information was recorded: the authors and publishing year, the study design and sample size, the key findings and conclusions, and the methodological quality and limitations. This approach allowed for a comprehensive and systematic analysis of the available literature.

Synthesis

The extracted data were synthesized qualitatively to provide a complete assessment of current information on the review topic. Where appropriate, a meta-analysis was performed to summarize the findings quantitatively.

Quality Assessment

The quality of the articles that made up the analysis was evaluated using known criteria, such as the Cochrane risk of bias tool for randomized controlled trials and the Newcastle-Ottawa Scale for observational studies. These assessments assessed if each study was of high, medium, or low quality.

Limitations

The review is subject to limitations, including potential publication bias and the variability in study designs and outcomes. These factors were considered when interpreting the results and drawing conclusions.

Ethical Considerations

As this is a review of previously published studies, no ethical approval was required. However, the review was conducted in accordance with the ethical standards of research integrity and reporting.

PRISMA Flowchart

**Figure 1:**
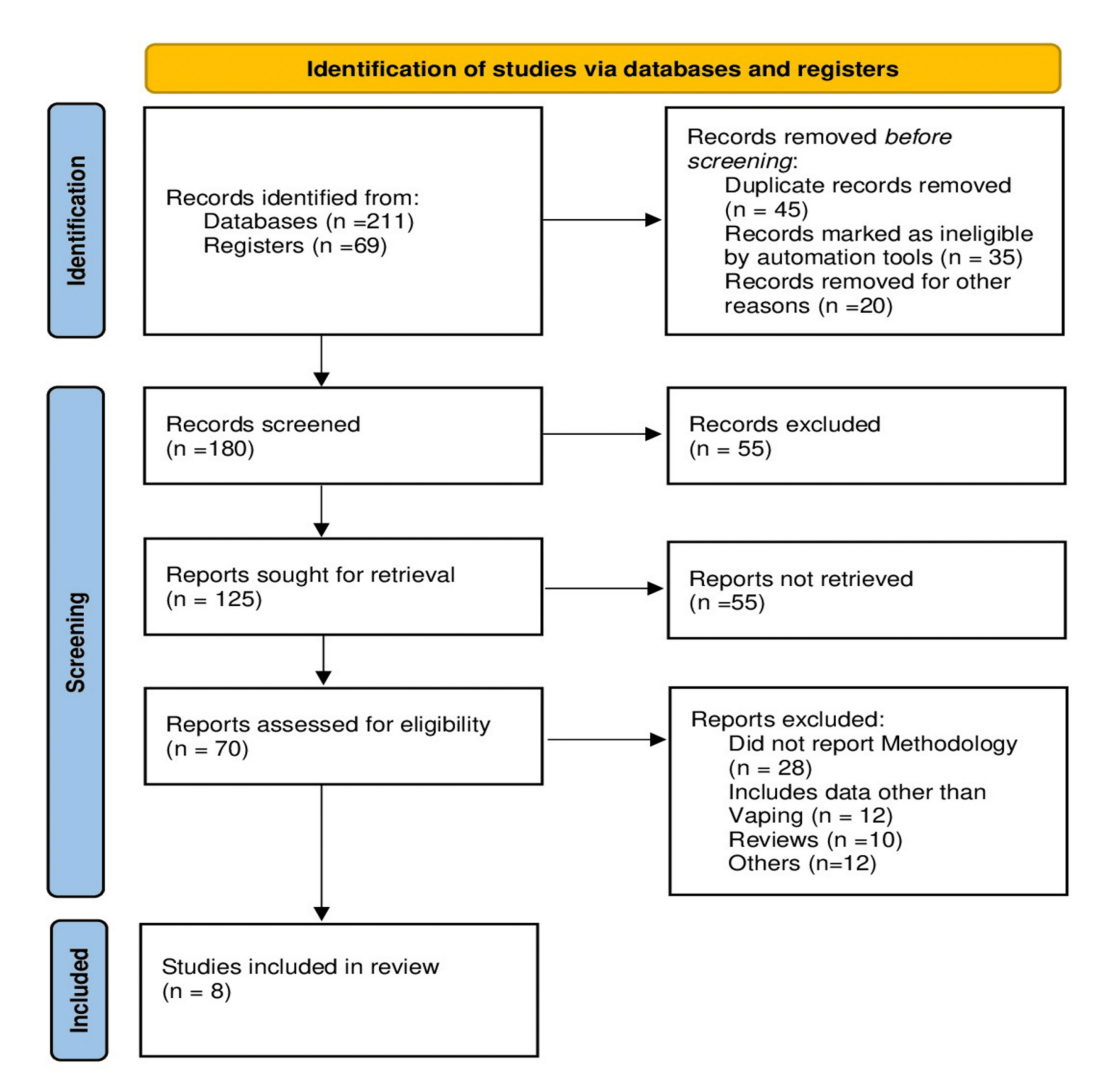
PRISMA flowchart of the study PRISMA: Preferred Reporting Items for Systematic reviews and Meta-Analyses

Figure 1 shows the Preferred Reporting Items for Systematic reviews and Meta-Analyses (PRISMA) flowchart illustrating the study selection process.

Components of a vape

A vaping device comprises several key components that work together to create the vaping experience. The drip tip at the end of the device facilitates the transfer of vaporized e-liquid from the coil to the user's lungs during inhalation [9]. The coil, composed of a cotton wick and a metal wire, absorbs, heats, and vaporizes the e-liquid, and its activation can be triggered by pressing a button or simply inhaling [10]. Positioned between the drip tip and the battery, the tank stores the e-liquid until the coil vaporizes it. The battery provides the necessary heat to the coil, enabling the production of e-liquid vapor [11]. Depending on the specific vaping device, the required charger type can vary, including options such as universal serial bus (USB) chargers, micro USB chargers, and cylindrical bay chargers. E-liquid, or vape juice or e-juice, is a mixture of PG, vegetable glycerin (VG), and optional nicotine, available in various strengths and ratios to cater to different preferences [12]. Those transitioning from smoking to vaping often start with nicotine-containing e-liquids and gradually decrease their nicotine intake until they are no longer using it [13].

Vaping-associated lung injury

EVALI has been connected to the occurrence of vitamin E acetate (VEA) and delta-9-tetrahydrocannabinol (THC) in both e-cigarettes and biological samples from those harmed. The current data are restricted because of the lack of established procedures for collecting data from patients and extensive physicochemical and toxicological research employing real-world vapor exposure. Thus, the specific pathophysiological process underlying EVALI remains unknown [14].

Patients with vaping-associated lung injury (VALI) usually present with a high body temperature, shortness of breath, an unproductive cough, chest discomfort, vomiting, nausea, and a bowel movement. On chest scans, all patients demonstrated bilateral infiltrates [15,16].

Histopathological examination of lung biopsies from individuals diagnosed with VALI shows signs of acute lung injury, including pneumocyte vacuolization and the presence of lipid-laden or foamy macrophages. All samples demonstrate a predominance of neutrophils [17,18].

The radiographic features of EVALI can vary, but several common findings are often present. On chest X-rays, bilateral infiltrates and ground-glass opacities, which appear as hazy areas that do not obscure the underlying lung structures, are frequently observed. Consolidation, where lung regions become filled with liquid instead of air, making them appear more solid, can also be seen. Computed tomography scans provide more detailed imaging and often reveal widespread ground-glass opacities involving multiple lobes, areas of consolidation, mosaic attenuation (a patchwork of different lung densities), pleural effusion (fluid accumulation between the layers of tissue lining the lungs and chest cavity), and septal thickening (thickening of the walls between the small air sacs in the lungs). These radiographic features are not specific to EVALI. They can be observed in many different lung diseases, so clinical correlation with patient history, including vaping habits, and additional diagnostic tests are necessary to confirm the diagnosis of EVALI [19-22].

Composition

The primary ingredients in e-liquids are PG, also called 1,2-propanediol, and glycerol, also known as glycerin or propane-1,2,3-triol. These substances act as humectants, ensuring that the e-liquid retains moisture and does not dry out [23]. The Food and Drug Administration has rated them as safe [24]. In fact, they are widely used in culinary and medicinal applications. An examination of 54 commercially available e-liquids indicated that PG and glycerol were present in almost all samples, with concentrations ranging from 0.4% to 98% (average 57%) for PG and 0.3% to 95% (average 37%) for glycerol [25]. Vegetable glycerin is a thicker, sweeter liquid that contributes to vapor production and provides a smoother throat hit than PG. It is derived from vegetable oils and adds viscosity to the e-liquid, enhancing the overall vaping experience [26]. Natural and artificial flavorings are food-grade additives that provide the distinct taste and aroma of the e-liquid. Flavorings can range from fruit and dessert flavors to menthol and tobacco blends. Manufacturers often use a combination of natural extracts and synthetic compounds to achieve specific flavor profiles [27]. E-liquids may contain varying levels of nicotine, usually expressed in milligrams per milliliter (mg/mL). Nicotine is an optional ingredient and can range from 0 mg/mL (nicotine-free) to 50 mg/mL or higher, depending on regional regulations and user preferences. Higher nicotine concentrations are commonly found in nicotine salt e-liquids, which are designed to deliver a smoother throat hit even at higher nicotine strengths [28]. Artificial sweeteners like sucralose or natural sweeteners like ethyl maltol are sometimes added to e-liquids to impart sweetness and balance flavor profiles [29]. The composition inside a vape device has been explained in Table 1.

**Table 1:** Comparison between a cigarette and a vape

Aspect	Cigarette	Vape
Primary components	Tobacco, paper, filter, and chemical additives	E-liquid ingredients (propylene glycol, vegetable glycerin, flavorings, and nicotine), battery, coil, and tank/cartridge
Method of use	Lit with a flame, produces smoke	Heated by a battery-powered coil, produces vapor
Nicotine delivery	Varies by cigarette type; generally delivers high nicotine levels	Adjustable nicotine levels in e-liquids; options from 0 mg/mL to high concentrations
Combustion	Involves combustion of tobacco, creating smoke and tar	No combustion; vaporizes e-liquid
Chemicals produced	Thousands of chemicals, including carcinogens like tar, formaldehyde, and benzene	Fewer chemicals; primarily propylene glycol, vegetable glycerin, and flavorings
Health risks	High risk of cancer, heart disease, respiratory issues, and other health problems	Generally considered less harmful than smoking; potential risks include lung irritation; long-term effects still under study
Secondhand exposure	Harmful secondhand smoke exposure	Less harmful secondhand vapor exposure, though not entirely risk-free
Odor	Strong, lingering odor	Usually mild and dissipates quickly
Regulation	Strictly regulated, with health warnings and advertising restrictions	Varies by region; increasing regulation on product safety, advertising, and sales
Cost	Ongoing cost of purchasing packs of cigarettes	Initial cost of device plus ongoing cost of e-liquids and replacement parts
User experience	Familiar, quick nicotine delivery	Customizable experience with various flavors, nicotine strengths, and device types
Environmental impact	Cigarette butts contribute to litter and pollution	E-waste from disposable cartridges, batteries, and devices

Commercial smokes include about 7,000 different chemicals. When these cigarettes are burnt, they may emit toxicants into mainstream smoke, sidestream smoke, secondhand smoke (SHS), thirdhand smoke, and discarded cigarette butts (CBs) [30]. A vape is made up of e-liquid (which contains PG, vegetable glycerin (VG), flavorings, and nicotine), a battery, a coil, and a tank or cartridge [31]. Despite these components, vaping is still considered a safer alternative to smoking cigarettes. Nicotine delivery varies by cigarette type, generally providing high levels of nicotine. In contrast, e-liquids offer adjustable nicotine levels, with options ranging from 0 mg/mL to high concentrations [32]. Traditional cigarettes involve the combustion of tobacco, producing smoke and tar [33]. In contrast, vaping does not involve combustion but vaporizes e-liquid. Cigarettes produce thousands of chemicals, including carcinogens such as tar, formaldehyde, and benzene. Vaping produces fewer chemicals, primarily consisting of PG, vegetable glycerin, and flavorings [34,35]. Smoking carries a high risk of cancer, heart disease, respiratory issues, and other health problems [36]. Vaping is generally considered less harmful than smoking, though potential risks include lung irritation, and the long-term effects are still under study [37,38]. Traditional cigarettes pose harmful risks from SHS exposure. Vaping, while less harmful than smoking, still presents secondhand vapor exposure, though not entirely risk-free. Smoking typically leaves a strong, lingering odor [39]. In contrast, vaping usually results in a mild odor that dissipates quickly. CBs contribute to litter and pollution, whereas vaping contributes to e-waste from disposable cartridges, batteries, and devices [40]. A comparison between cigarettes and vaping is shown in Table 2.

**Table 2:** Composition inside a vape PG: propylene glycol; VG: vegetable glycerin

Component	Description	Reference
PG	Clear, odorless liquid; carrier for flavorings; and provides a throat hit	Stefaniak et al. [24]
VG	Thicker, sweeter liquid; enhances vapor production; and smoother throat hit than PG	Hahn et al. [26]
Flavorings	Natural or artificial additives; and provide taste and aroma (e.g., fruit, dessert, and menthol)	Kim et al. [27]
Nicotine	Optional ingredient; varies in strength (0 mg/mL to high concentrations for nicotine salts); and provides nicotine delivery	Kennedy et al. [28]
Additives	Various additives like water, alcohol, and sweeteners to adjust viscosity, enhance flavor, and add sweetness	Grilo et al. [29]

Recent studies have highlighted significant concerns regarding vaping-related health impacts. Salzman et al. reviewed the outbreak of EVALI, emphasizing its rapid rise and severe respiratory symptoms associated with vaping, underscoring EVALI as a pressing public health issue [1]. McDonough et al. explored biomarkers of exposure and toxicity in e-cigarette users and EVALI, advancing diagnostics and treatment understanding [6]. Ghinai et al. investigated EVALI cases in Illinois and Wisconsin, linking many instances to THC-containing products, particularly those with VEA [10]. Ali et al. presented a case series demonstrating severe lung injuries linked to vaping, stressing the need for awareness, and caution [15]. Brown and Cheng characterized e-cigarette products, highlighting variations in design and nicotine delivery systems crucial for user experience [23]. Stefaniak et al. reviewed toxicological studies on flavoring and cannabis in e-liquids, identifying potential health risks [24]. Khlystov and Samburova analyzed toxic aldehyde emissions from flavored e-cigarettes, advocating for regulation to mitigate health risks [32]. Chakma et al. discussed India's ban on e-cigarettes, supporting it as a critical public health measure despite enforcement challenges [39].

The objectives, methodologies, key findings, and conclusions of several studies have been thoroughly examined and detailed. These studies encompass a range of research goals and employ various approaches to achieve their aims. Key findings from these studies provide valuable insights, and the conclusions drawn highlight their significance and implications. A comprehensive summary of these elements for each study can be found in Table 3.

**Table 3:** Summary of key studies on vaping and e-cigarette-related health issues EVALI: e-cigarette or vaping use-associated lung injury; e-cigarette: electronic cigarette; THC: tetrahydrocannabinol

Author(s)	Year	Title	Journal	Objective	Methodology	Key findings	Conclusion
Salzman et al. [1]	2019	Vaping associated lung injury (EVALI): an explosive United States epidemic	Mo Med	To review the outbreak of EVALI and its clinical presentation and management	Review of clinical cases and literature	Rapid rise in cases of EVALI, characterized by severe respiratory symptoms linked to vaping	EVALI represents a significant public health concern requiring immediate attention
McDonough et al. [6]	2021	Recent updates on biomarkers of exposure and systemic toxicity in e-cigarette users and EVALI	Am J Physiol Lung Cell Mol Physiol	To review recent updates on biomarkers of exposure and systemic toxicity in e-cigarette users and EVALI	Review of current literature and studies	Identification of biomarkers related to exposure and systemic toxicity, implications for EVALI diagnosis, and management	Advances in biomarker research provide better understanding of e-cigarette exposure and EVALI, aiding in diagnosis and treatment
Ghinai et al. [10]	2019	E-cigarette product use, or vaping, among persons with associated lung injury	MMWR Morb Mortal Wkly Rep	To investigate the characteristics of EVALI cases in Illinois and Wisconsin	Epidemiological study, data analysis of EVALI cases	Majority of cases associated with THC-containing products, particularly those with vitamin E acetate	Highlighted the link between THC-containing vaping products and the outbreak of lung injury
Ali et al. [15]	2020	A case series of vaping-induced lung injury in a community hospital setting	Case Rep Pulmonol	To present a case series of vaping-induced lung injury in a community hospital	Retrospective case series	Cases of severe lung injury associated with vaping, diverse clinical presentations	Vaping can lead to serious lung injury requiring medical attention, emphasizing the need for awareness and caution
Brown and Cheng [23]	2014	Electronic cigarettes: product characterisation and design considerations	Tob Control	To characterize electronic cigarette products and discuss design considerations	Product analysis and review	Variation in product design and nicotine delivery systems	Design plays a crucial role in the effectiveness and user experience of electronic cigarettes
Stefaniak et al. [24]	2021	Toxicology of flavoring- and cannabis-containing e-liquids used in electronic delivery systems	Pharmacol Ther	To investigate the toxicology of flavoring- and cannabis-containing e-liquids used in e-cigarettes	Review of toxicological studies	Identified harmful effects of various flavoring and cannabis compounds in e-liquids	Highlighted the potential risks associated with the use of flavoring and cannabis compounds in e-liquids
Khlystov and Samburova [32]	2016	Flavoring compounds dominate toxic aldehyde production during e-cigarette vaping	Environ Sci Technol	To analyze toxic aldehyde production during e-cigarette vaping	Laboratory study, analysis of aerosol samples	Flavoring compounds contribute significantly to toxic aldehyde emissions during vaping	Emphasized the importance of regulating flavoring compounds in e-cigarettes to reduce health risks
Chakma et al. [39]	2020	The e-cigarettes ban in India: an important public health decision	Lancet Public Health	To discuss the public health implications of the e-cigarettes ban in India	Literature review and analysis	Support for the e-cigarettes ban in India, citing potential health benefits and regulatory challenges	Ban on e-cigarettes in India is a significant public health measure, despite challenges in enforcement and regulation

## Conclusions

In conclusion, this systematic literature review provides a comprehensive examination of the composition, health impacts, and regulatory dynamics of e-cigarettes. The components of e-cigarettes, including PG, vegetable glycerin, nicotine, and flavorings, are explored in depth to understand their roles and potential health implications. While e-cigarettes are often marketed as a safer alternative to traditional tobacco products, this review highlights that they are not without risks, particularly concerning respiratory and cardiovascular health. Incidents like the EVALI outbreak underscore the need for ongoing research to fully understand the long-term effects of vaping.

E-cigarettes have complex health impacts. While they might be less harmful than traditional cigarettes, evidence shows they still pose significant risks, such as EVALI. Public awareness and education about vaping dangers are crucial. Strong regulatory efforts are needed to prevent youth use and manage marketing tactics targeting young people. Ongoing research and vigilant oversight are essential. Regulators face the challenge of balancing harm reduction for adult smokers with protecting youth health. Future studies should investigate the long-term effects and effectiveness of e-cigarettes as cessation tools to inform policy and public health measures, ensuring benefits while minimizing harms.
